# Free water modeling of peritumoral edema using multi-fiber tractography: Application to tracking the arcuate fasciculus for neurosurgical planning

**DOI:** 10.1371/journal.pone.0197056

**Published:** 2018-05-10

**Authors:** Shun Gong, Fan Zhang, Isaiah Norton, Walid I. Essayed, Prashin Unadkat, Laura Rigolo, Ofer Pasternak, Yogesh Rathi, Lijun Hou, Alexandra J. Golby, Lauren J. O’Donnell

**Affiliations:** 1 Brigham and Women’s Hospital, Harvard Medical School, Boston, Massachusetts, United States of America; 2 Department of Neurosurgery, Shanghai Institute of Neurosurgery, Shanghai Changzheng Hospital, Shanghai, China; University of North Carolina at Chapel Hill, UNITED STATES

## Abstract

**Purpose:**

Peritumoral edema impedes the full delineation of fiber tracts due to partial volume effects in image voxels that contain a mixture of cerebral parenchyma and extracellular water. The purpose of this study is to investigate the effect of incorporating a free water (FW) model of edema for white matter tractography in the presence of edema.

**Materials and methods:**

We retrospectively evaluated 26 consecutive brain tumor patients with diffusion MRI and T2-weighted images acquired presurgically. Tractography of the arcuate fasciculus (AF) was performed using the two-tensor unscented Kalman filter tractography (UKFt) method, the UKFt method with a reduced fiber tracking stopping fractional anisotropy (FA) threshold (UKFt+rFA), and the UKFt method with the addition of a FW compartment (UKFt+FW). An automated white matter fiber tract identification approach was applied to delineate the AF. Quantitative measurements included tract volume, edema volume, and mean FW fraction. Visual comparisons were performed by three experts to evaluate the quality of the detected AF tracts.

**Results:**

The AF volume in edematous brain hemispheres was significantly larger using the UKFt+FW method (p<0.0001) compared to UKFt, but not significantly larger (p = 0.0996) in hemispheres without edema. The AF size increase depended on the volume of edema: a significant correlation was found between AF volume affected by (intersecting) edema and AF volume change with the FW model (Pearson r = 0.806, p<0.0001). The mean FW fraction was significantly larger in tracts intersecting edema (p = 0.0271). Compared to the UKFt+rFA method, there was a significant increase of the volume of the AF tract that intersected the edema using the UKFt+FW method, while the whole AF volumes were similar. Expert judgment results, based on the five patients with the smallest AF volumes, indicated that the expert readers generally preferred the AF tract obtained by using the FW model, according to their anatomical knowledge and considering the potential influence of the final results on the surgical route.

**Conclusion:**

Our results indicate that incorporating biophysical models of edema can increase the sensitivity of tractography in regions of peritumoral edema, allowing better tract visualization in patients with high grade gliomas and metastases.

## Introduction

The use of preoperative imaging for neurosurgical planning is increasing, with the aim of preserving neurological function and precisely removing lesions [1–3]. The addition of diffusion magnetic resonance imaging (dMRI), the only technique for identifying white matter microstructure *in vivo*, has been shown to increase extent of tumor resection and improve survival [4,5]. Tractography, derived from dMRI, has enabled evaluation and three-dimensional visualization of critical fiber tracts. However, with increasing clinical use of tractography for white matter fiber tracking, challenges such as peritumoral edema, crossing fibers, displacement, infiltration, and the variable combination of these effects are increasingly encountered using conventional tractography techniques available to clinicians [6,7]. Commercial clinical software primarily relies on the diffusion tensor, which can model a single fiber per voxel, while many advanced research tractography techniques that can model crossing fibers are entering the clinical realm [8].

When tracking in the vicinity of brain tumors, peritumoral edema is one particular challenge that limits accurate brain tumor resection [9,10]. This vasogenic edema consists of extracellular free water (FW) resulting from blood brain barrier breakdown leaking fluid into the extracellular space [11]. It is a challenge to detect white matter microstructure when image voxels include partial volume of FW [12]. Our previous work has shown that two-tensor unscented Kalman filter tractography (UKFt) [13], a multi-fiber tractography method, could achieve more satisfactory tractography results than the clinical standard of single-tensor tractography in the presence of crossing fibers and edema [14,15]. In addition, we have shown the potential of an automated method for identifying fiber tracts of interest for neurosurgical planning, even in patients with mass lesions and edema [16].

However, the effects of peritumoral edema still impede the full delineation and correct identification of fiber tracts. In particular, clinical strategies to enable tracking through edema involve patient-specific and time-consuming interactive adjustment of various fiber tracking thresholds that are used to start and stop tracking. Early investigations demonstrated that lowering the fractional anisotropy (FA) threshold could enable increased tracking in edema and tumors [10,17,18]. Today, each state-of-the-art multi-fiber tractography method relies on a different threshold, which is necessarily specific to the fiber model and tractography framework (e.g. fiber orientation distribution (FOD) based thresholds, apparent fiber density, generalized anisotropy, bundle-specific thresholds, and free-water corrected thresholds) [12,13,19–21]. We have demonstrated that varying tractography thresholds has a large impact on fiber tracking using UKFt near edema, but multiple thresholds can interact and their impact differs across patients and tracts [22]. Relying on user interaction to set such patient-specific, tract-specific, and tumor-specific threshold parameters is not practical in a scenario of automated tractography, where the goal is to reduce time-consuming user interaction and the known operator bias that leads to variability across expert raters and across tractography methods [23,24].

One possible way to address the challenge of tracking through edema, without requiring an operator to select patient-specific thresholds, is by modeling the edema separately from the fiber tract. This modeling strategy can potentially have the effect of an adaptive local threshold that adjusts for the presence of edema. FW is defined as self-diffusing water molecules that do not experience restriction or hindrance from their surroundings during the time of the diffusion MRI experiment [12,25,26]. In typical experiments the diffusion time is a few tens of milliseconds, which means that in brain scans FW can only be measured in the relatively large water compartment of the extracellular space. FW imaging is an analysis method for dMRI data that separately models the contribution of extracellular FW and water that is in the vicinity of cellular tissue. In FW imaging, the FW is explicitly modeled by an isotropic (spherical) tensor with diffusivity fixed to that of FW. Using a FW model (also called FW elimination) increases the precision of conventional metrics such as fractional anisotropy (FA) and trace, and quantitatively estimates the degree of vasogenic edema and potentially neuroinflammation [27,28]. For example, recent studies applying FW to diffusion tensor imaging (DTI) data have shown improved sensitivity of DTI-based metrics in major depressive disorder [29], association of state and trait delusions in chronic schizophrenia with microstructural processes [30], and increased FW values in the posterior substantia nigra in Parkinson’s disease [31]. While these and other studies have shown the advantages of FW in general neuroimage analysis, Pasternak et al. and Lecoeur et al. have applied FW to study peritumoral edema. They found that peritumoral edema had high FW volume where the fiber tracts terminated [12,26] and tractography performance increased in an initial test of a FW model in five patients with brain tumors [32,33]. Recently, we demonstrated that UKFt plus a FW model could recover fiber tracts in a synthetic edema phantom, but the effect of including FW was not conclusive in our small study of two patients with brain tumors [22]. Therefore, the potential advantages of FW in tractography of neurosurgical patients with brain tumors are still not clear.

In this study, we investigate the addition of a FW model in tracing the arcuate fasciculus (AF) through regions of peritumoral edema. The AF is a language tract widely considered to be important for neurosurgical planning. Comparison on a consecutive retrospective series of 26 brain tumor patients was used to evaluate the performance of a FW model in the UKFt method. While FW modeling has been used recently in tractography in multiple neuroscientific studies [29–31,34–38], to our knowledge this is the first study applying the FW model to a cohort of patients with brain tumors.

## Materials and methods

### 2.1. Data acquisition

For this study, we retrospectively evaluated consecutive brain tumor patients who had undergone dMRI and T2-weighted images acquired presurgically at Brigham and Women’s Hospital, Boston, USA. A total of 26 consecutive brain tumor patients (15 male, 11 female; age range 23–72 years) scanned with a diffusion imaging sequence were included in this study. All images were obtained using Siemens 3T scanners (Siemens Trio and Verio, Siemens Healthcare, Erlangen, Germany) equipped with a 12-channel head coil. T2-weighted scans (TR = 7500 ms, TE = 30 ms, matrix = 512 × 512, FOV = 25.6 cm, flip angle = 20°, 176 slices, voxel size = 0.5 × 0.5 × 1 mm^3^) were acquired as clinically indicated for each patient. Diffusion weighted images (DWI) were acquired using an echo planar imaging (EPI) sequence (30 gradient directions, 1 baseline (b = 0) image, b = 2000 s/mm^2^, TR = 12700 ms, TE = 98 ms, flip angle = 90°, matrix = 100 × 90, FOV = 22 cm, 59 axial slices, voxel size = 2.3 x 2.3 x 2.3 mm^3^). Detailed patient demographics, including tumor histology, tumor location and edema location, are listed in Table 1. The study was approved by the Partners Healthcare Institutional Review Board, and written informed consent was obtained from all subjects prior to participation.

**Table 1:** Patient demographics.

Patient	Age	Sex	Tumor type	Tumor Location	Edema Location
P1	28	F	Oligodendrioma, WHO II	Temporal (L)	-
P2	34	F	Recurrent metastatic carcinoma	Frontal (L)	Frontal-parietal (L)
P3	57	M	Glioblastoma, WHO IV	Temporal (R)	Temporal-occipital (R)
P4	66	F	Glioblastoma, WHO IV	Temporal (L)	Temporal (L)
P5	63	M	Metastatic melanoma	Temporal-frontal (R)	Temporal-frontal-parietal-occipital (R)
P6	52	F	Metastatic carcinoma	Temporal-frontal (R)	Temporal-frontal-parietal-occipital (R)
P7	70	M	Anaplastic astrocytoma, WHO III	Temporal-insular (L)	-
P8	26	F	Anaplastic astrocytoma, WHO III	Frontal (L)	-
P9	57	F	Diffuse astrocytoma, WHO II	Frontal (R)	-
P10	59	F	Low grade glial/glioneuronal tumor	Frontal (L)	-
P11	57	M	Glioblastoma, WHO IV	Temporal (L)	Temporal-insular-parietal-occipital (L)
P12	52	M	Malignant spindle cell neoplasm	Frontal-parietal (B)	Frontal-parietal (B)
P13	51	F	Glioblastoma, WHO IV	Parietal (L)	Parietal (L)
P14	51	M	Glioblastoma, WHO IV	Temporal-parietal-frontal (R)	Temporal-parietal-frontal (R)
P15	38	M	Anaplastic astrocytoma, WHO III	Temporal-insular-frontal (R)	Temporal-insular-parietal-frontal (R)
P16	70	F	Glioblastoma, WHO IV	Frontal-insular (L)	Frontal-insular-temporal-parietal (L)
P17	23	M	Anaplastic astrocytoma, WHO III	Frontal (R)	-
P18	34	F	Diffuse astrocytoma, WHO II	Frontal (R)	-
P19	37	M	Glioblastoma, WHO IV	Frontal-parietal (R)	Frontal-parietal (R)
P20	61	M	Glioblastoma, WHO IV	Frontal-parietal (L)	Frontal-occipital-temporal-parietal (L)
P21	56	M	Angiomatous Meningioma, WHO I	Frontal-parietal (R)	Temporal-frontal-parietal (R)
P22	54	F	Metastatic melanoma	Frontal-parietal (L)	Frontal-insular-temporal-parietal (L)
P23	54	M	Meningioma, WHO I	Frontal-parietal (R)	-
P24	26	M	Recurrent Glioblastoma, WHO IV	Frontal (L)	Frontal-insular (L)
P25	36	M	Recurrent Oligodendroglioma	Parietal (L)	Parietal (L)
P26	72	M	Metastatic melanoma	Occipital (L)	Frontal-occipital-temporal-parietal (B)

P, patient; F, female; M, male; WHO, World Health Organization; L, left; R, right; B, bilateral.

### 2.2. Data preprocessing

We used DWIConvert (github.com/BRAINSia/BRAINSTools) for conversion from DICOM and DTIPrep (www.nitrc.org/projects/dtiprep) [39] for motion and eddy current distortion correction. Then the 3D Slicer (www.slicer.org) [40,41] SlicerDMRI extension (dmri.slicer.org) [42] was applied to obtain baseline images (B0, the b = 0 image from the DWI) and derive binary brain masks from the DWI images. A rigid registration was computed between the baseline image and the T2 image in 3D Slicer using the *General Registration (BRAINS) module*. We applied this rigid registration later to the fibers for visualization in anatomical T2 space. For visualization purposes, DTI images were estimated from the DWI data and directionally encoded color FA maps were calculated in 3D Slicer using the *Diffusion Tensor Estimation module*.

### 2.3. Seeding of tractography

The FW model is implemented in the open-source UKFt software package (github.com/pnlbwh/ukftractography) [13,15,43]. The UKFt method traces local fiber orientations using the model estimation at previous positions to guide the estimation at the current position. In contrast to other methods that fit a model to the signal independently at each voxel [44], in the UKF framework [45] each tracking step employs prior information from the previous step to help stabilize model fitting. Two models were studied in this paper: the two-tensor UKFt model consists of two cylindrically symmetric tensors, while the two-tensor UKFt with FW model adds a third, isotropic, tensor with diffusivity equal to that of free water. Whole-brain tractography was performed using the two-tensor UKFt and two-tensor UKFt with FW model (UKFt+FW) methods in the same dataset.

We used default values for the UKFt seeding and stopping FA thresholds, where these defaults have previously been empirically determined across multiple datasets [13–16]. Tractography was seeded with 20 seeds per voxel in all voxels within the binary brain mask where FA was greater than 0.18 (default). We have previously shown that a dense seeding of 20 seeds per voxel is a good value for successful identification of the arcuate fasciculus and other tracts in our brain tumor patient data [16]. Tracking stopped where the FA (of the tensor being tracked) value fell below 0.15 (default) or the normalized average signal (the sum of the normalized signal across all gradient directions) fell below 0.08. The normalized average signal measure was employed to robustly distinguish between white/gray matter and cerebrospinal fluid (CSF) regions. The normalized average signal threshold was reduced below the default value in patient data to enable higher sensitivity for tracking in or near edema. In the UKFt+FW method, detailed parameter settings were the same as UKFt, with the addition of the free water model. The FW model estimated the fractional volume of the FW compartment (the FW fraction), yielding diffusion tensors that were corrected for the contribution of FW.

To investigate the influence of stopping thresholds on increased tracking in edema and tumors, for each patient we generated an additional 6 tractography datasets by reducing the stopping FA threshold (from the default 0.15 to a very low setting of 0.03). To enable a fair comparison of FA reduction and FW modeling, across these multiple FA thresholds we chose the setting that generated an AF tract (see Section 2.4 for identification of AF) with the most similar volume (see Section 2.6 for volume measurement) to that generated using the UKFt+FW method. In the rest of the paper, we refer this method to as UKFt+rFA. Therefore, in total, for each case, we obtained three tractography datasets generated using UKFt, UKFt+FW and UKFt+rFA, respectively.

### 2.4. Automatic identification of AF

**Fig 1:**
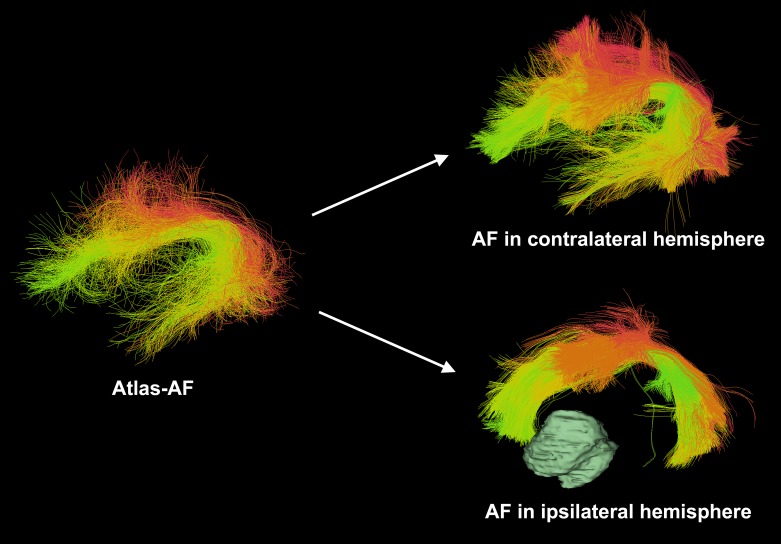
Automated white matter fiber tract identification of the arcuate fasciculus (AF) tracts in patient data. Visualization of AF tracts uses colors indicating individual fiber clusters as defined in the atlas, where each fiber cluster has a unique color, and similar clusters have similar colors (green surface model shows the brain tumor).

Our approach followed an automated white matter fiber tract identification method that we published recently for neurosurgical planning [16]. After performing whole-brain tractography using the UKFt and UKFt+FW methods, fiber clusters were automatically parcellated using fiber tract registration and clustering methods [46,47]. Patient-specific bilateral AF clusters (18 clusters) were identified according to a fiber cluster atlas, which was previously created with a data-driven machine learning method to describe common white matter anatomy across multiple subjects [16]. Briefly, to cluster using the atlas, each fiber is compared to multiple fibers in the atlas, giving a feature vector or “fingerprint” that is used to classify the fiber into a cluster. This process uses spectral embedding [48] for robust representation of fibers based on their similarities to other fibers. The fiber similarity relationships can be visualized using colors derived from the spectral embedding [46,49], which allows us to automatically assign a color to each fiber cluster in the atlas. Fiber tracts were visualized using fiber cluster colors from the atlas, where each cluster has a unique color, and similar clusters have similar colors (Fig 1). All software used is publicly available, including computational tractography analysis methods [46,47] (github.com/SlicerDMRI/whitematteranalysis), and tractography visualization with anatomical hierarchies in 3D Slicer via the SlicerDMRI project (dmri.slicer.org).

### 2.5. Segmentation of edema

To evaluate the presence of edema, we segmented edematous brain regions using T2-weighted images in 3D Slicer. We manually delineated the outline of edema and tumor according to the bright signals in anatomical T2 space using the 3D Slicer *Editor module* and reconstructed the edema (transparent blue) and tumor (green) models using the *Model Maker module*. The segmentation results were checked by multiple expert readers (SG, WIE, PU) using T1/T2-weighted images and fluid-attenuated inversion recovery images.

### 2.6. Volume measurement of the AF

The voxel volume of the AF was calculated as follows. We used the *Tractography to Mask Image module* in 3D Slicer to transform the AF into a label map (a segmented image volume, in this case a binary mask) in T2 space. This module sets a specified label value in the label map at every vertex of each of the fibers in each AF. Then, we applied the *Label Statistics module* to calculate the label volume. The volume of the AF was defined as the volume of the voxels occupied by the fibers in each subject [50]. We then identified the overlapping voxels containing edema and AF using the *Simple Filters module* and measured this volume as above to obtain the volume of AF traced within the edema. To provide additional information regarding volume changes, we also measured the number and length (in mm) of fibers in the AF.

### 2.7. FW fraction measurement

In the edematous hemispheres, the AF was split into two parts: fibers that traversed edema and those that did not, using the *Tractography ROI Selection module* in 3D Slicer. The FW fraction of each part was measured separately using the *Tractography Measurements module*.

### 2.8. Statistical analysis

We performed statistical analysis using GraphPad Prism (version 7.0a; GraphPad Software, Inc.) and Matlab (version R2015a, The MathWorks, Inc). Standard summary statistics were used to describe the measurement data including the volume, number, length and FW fraction of fibers in the AF. Then we used paired t-tests to compare means across models employed in tractography (UKFt vs UKFt+FW). A value of p<0.05 (two-tailed) was considered statistically significant.

### 2.9. Expert judgments

In addition to the above quantitative comparisons, we performed an expert judgment experiment to evaluate the quality of the AF tracts obtained in the different methods (UKFt, UKFt+rFA and UKFt+FW). The five patient datasets with the smallest AF volumes among the 20 edematous hemispheres were selected to perform this experiment, because in these patients the AF tracts were the most affected by the edema and thus were the most crucial ones to inspect the methods’ performance in tracing through the edema. Three experts (I.N., W.I.E. and P.U.) visually ranked the three AF tracts obtained from each patient, as follows. The three tracts were loaded into the 3D Slicer software, overlaid on the anatomical T2 image. Segmentations of the tumor and edema were provided to show the relative positions of the tracts to the tumor and edema. Raters were blinded to the origin of each tract: the three tract filenames and their display orders in the 3D Slicer were totally randomized. In this way, there was no information about which tract was from which method.

The overall tracts quality grading was performed following standard anatomical knowledge and according to the potential influence of the final results on the surgical route. Results that showed the most successful identification of the AF tract near lesions or edema, while avoiding false positive tracking, received the best grades. Each expert was asked to rank the three tracts based on their judgment, where a rank of 1 was the best and 3 was the worst. There could be two tracts that were equally good or bad to the experts. In such a situation, these two tracts obtained the same rank score. This process provided a total of 15 ranking scores for each method (5 patients times 3 raters). To summarize the expert judgment results, for each method, we then computed the mean and the standard deviation of the ranking scores.

## Results

The fiber tractography performed using the UKFt and UKFt+FW methods was compared visually and quantitatively. 18 of 26 patients had peritumoral edema, in which a total of 20 hemispheres contained edema (2 patients had edema in both hemispheres). We focused on the AFs located in the edematous hemispheres.

### 3.1. Case illustrations

**Fig 2:**
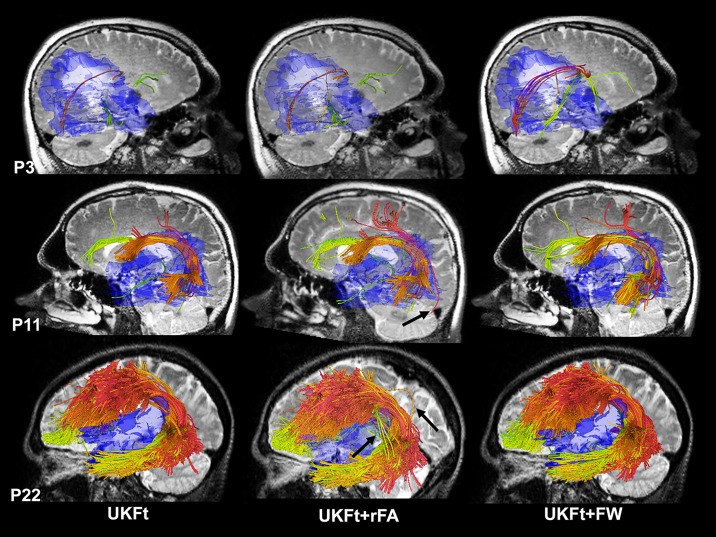
Automatically detected arcuate fasciculus tract clusters in example datasets from patients with edema. Views are from left or right based on the involvement with edema, using the UKFt, UKFt+rFA and UKFt+FW methods of P3, P11, P22. A T2-weighted image is shown behind the fiber tracts. Edema is shown in transparent blue. Tract colors indicate individual fiber clusters as defined in the atlas. For the UKFt+rFA method, the FA thresholds were set to 0.03, 0.07 and 0.07 for the three cases, respectively, to achieve the most similar AF volumes to those obtained using the UKF+FW method. Overall, reducing the FA threshold and adding the FW model both resulted in visually larger AF tracts on the three cases compared to UKFt. In P3, the UKFt+FW method obtained more visually apparent anatomically correct AF fibers (red). In P11 and P22, while tracts obtained using the UFKt+rFA and UKFt+FW methods are visually similar, the UKFt+rFA method introduced more visually apparent false positive fibers (as indicated by the black arrows) than the UKFt+FW method.

**Fig 3:**
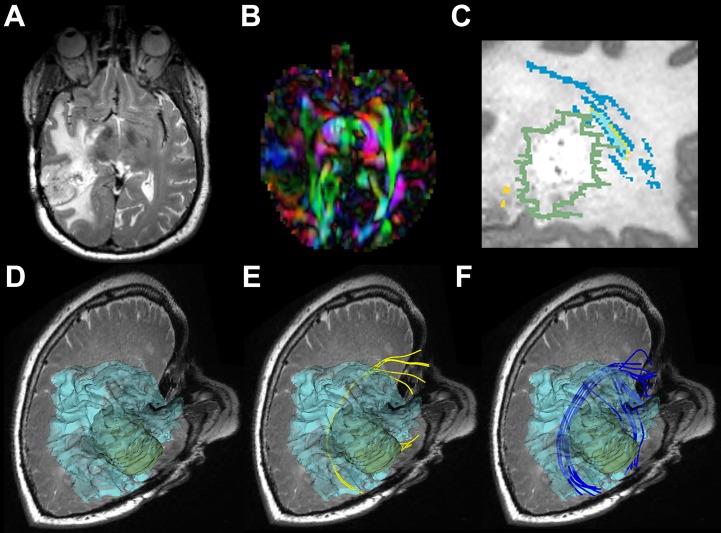
Images from P3 with a right temporal lesion. (a) T2-weighted image shows a temporal tumor with extensive edema around the lesion. (b) Directionally encoded color FA map shows reduced anisotropy in AF. (c) Magnified sagittal view of a T2-weighted image with overlaid label maps showing fibers reconstructed by the UKFt (yellow) and UKFt+FW (dark blue) methods and where they overlap (light blue) near the tumor (green outlined). (d) The reconstructed edematous area (transparent blue surface model) near the tumor (green model). (e) The AF using the UKFt method displayed in yellow. (f) The AF using the UKFt+FW method displayed in blue.

**Fig 4:**
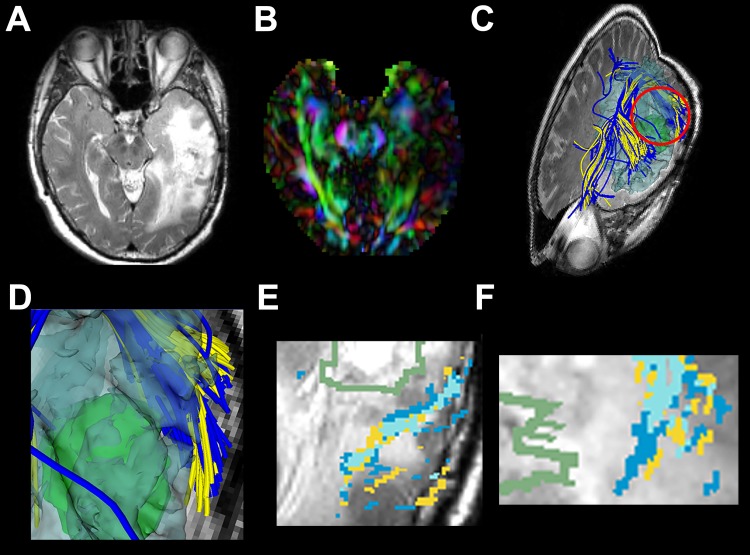
Images from P11 with a left temporal GBM. (a) T2-weighted image shows a temporal tumor with extensive edema around the lesion. (b) Directionally encoded color FA map shows reduced anisotropy in AF. (c) The AF is reconstructed by the UKFt (yellow tracts) and UKFt+FW (blue tracts) methods. (d) The magnified red circle (in c) area shows a slight difference in fibers traced using the UKFt+FW vs the UKFt method near the tumor (green surface model). (e) Magnified axial view of a T2-weighted image with overlaid label maps showing fibers reconstructed by the UKFt (yellow) and UKFt+FW (dark blue) methods and where they overlap (light blue) near the tumor (green outlined). (f) Magnified sagittal view of a T2-weighted image with overlaid label maps showing fibers reconstructed by the UKFt and UKFt+FW methods and where they overlap near the tumor.

**Fig 5:**
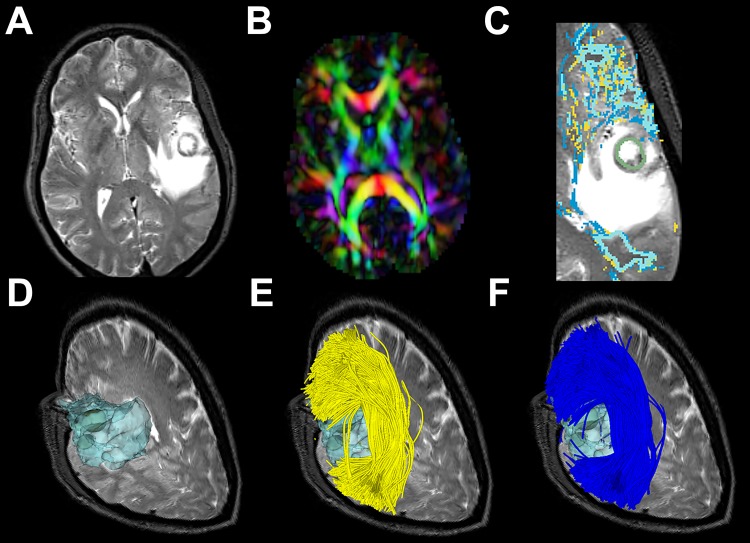
Images from P22 with a left frontal-parietal lesion. (a) T2-weighted image shows a temporal tumor with extensive edema around the lesion. (b) Directionally encoded color FA map shows reduced anisotropy in AF. (c) Magnified axial view of a T2-weighted image with overlaid label maps showing fibers reconstructed by the UKFt (yellow) and UKFt+FW (dark blue) methods and where they overlap (light blue) near the tumor (green outlined). (d) The reconstructed edematous area (transparent blue surface model) near the tumor (green model). (e) The AF using the UKFt method displayed in yellow. (f) The AF using the UKFt+FW method displayed in blue.

Fig 2 shows results in three cases that illustrate a range of edema involvement of the AF. In case P3, the right AF is almost entirely within the edematous region, while in case P11, part of the left AF is located within the edematous region. In P22, the edema intersects a small part of the left AF. Figs 3 to 5 show more details of these three cases comparing UKF and UKF+FW in patient datasets.

#### 3.1.1. Patient 3

Fig 3 shows images from patient 3 (P3) who had glioblastoma in right temporal lobe and peritumoral edema in right temporal and occipital lobe around the tumor (Fig 3a and 3d). The axial view of the directionally encoded color FA map (Fig 3b) (with blue indicating superior-inferior, red indicating transverse, and green indicating anterior-posterior DTI eigenvector orientations) demonstrated reduced anisotropy in the tumor hemisphere when compared with the healthy hemisphere. The overlaid label maps (Fig 3c) show fibers reconstructed by the UKFt (yellow) and UKFt+FW (dark blue) methods and where they overlapped (light blue) near the tumor (green outlined). Relatively small AF fiber bundles were traced using both the UKFt and UKFt+FW methods, but the UKFt+FW method traced more AF fibers (dark blue label) traversing the edema. Few fibers were traced near the tumor using the UKFt method (Fig 3e), while more fibers were traced projecting to the superior and inferior temporal gyri through the edematous area using the UKFt+FW method (Fig 3f). The total volume of the AF tract was 972 mm^3^ and 1,932 mm^3^ using the UKFt and UKFt+FW methods, respectively. The volume of the tract that intersected the edema was 246.9 mm^3^ and 867.7 mm^3^ using the UKFt and UKFt+FW methods, respectively.

#### 3.1.2. Patient 11

Fig 4 shows images from patient 11 (P11) who had glioblastoma in the left temporal lobe and peritumoral edema in the left temporal, insular, parietal and occipital lobes around the tumor (Fig 4a). The axial view of the directionally encoded color FA map (Fig 4b) demonstrated reduced anisotropy in AF when compared with the healthy hemisphere. In this patient, we found that the AF displayed more fibers than P3 both using the UKFt and UKFt+FW methods, and that the UKFt+FW method provided the ability to trace the AF slightly nearer to the tumor (Fig 4c and 4d). The overlaid label maps (Fig 4e and 4f) also showed the UKFt+FW method traced the AF (dark blue label) slightly nearer to the tumor. The total volume of the AF tract was 10,167 mm^3^ and 12,971 mm^3^ using the UKFt and UKFt+FW methods, respectively. The volume of the tract that intersected the edema was 3,742.1 mm^3^ and 4,081.7 mm^3^ using the UKFt and UKFt+FW methods, respectively.

#### 3.1.3. Patient 22

Fig 5 shows images from patient 22 (P22) who had metastatic melanoma in left frontal-parietal lobe and peritumoral edema in left frontal, insular, temporal and parietal lobes around the tumor (Fig 5a and 5d). The axial view of the directionally encoded color FA map (Fig 5b) demonstrated reduced anisotropy in AF when compared with the healthy hemisphere. The overlaid label maps (Fig 5c) showed that the UKFt+FW method traced the AF very similarly to the UKFt method, but with a subtle increase in fibers traced through edema and near the tumor. Overall, temporal and frontal fibers were similarly traced using the UKFt (Fig 5e) and UKFt+FW (Fig 5f) methods. The total volume of the AF tract was 71,476 mm^3^ and 84,254 mm^3^ using the UKFt and UKFt+FW methods, respectively. The volume of the tract that intersected the edema was 1,641.6 mm^3^ and 2,854.3 mm^3^ using the UKFt and UKFt+FW methods, respectively.

### 3.2. Volume analysis

**Fig 6:**
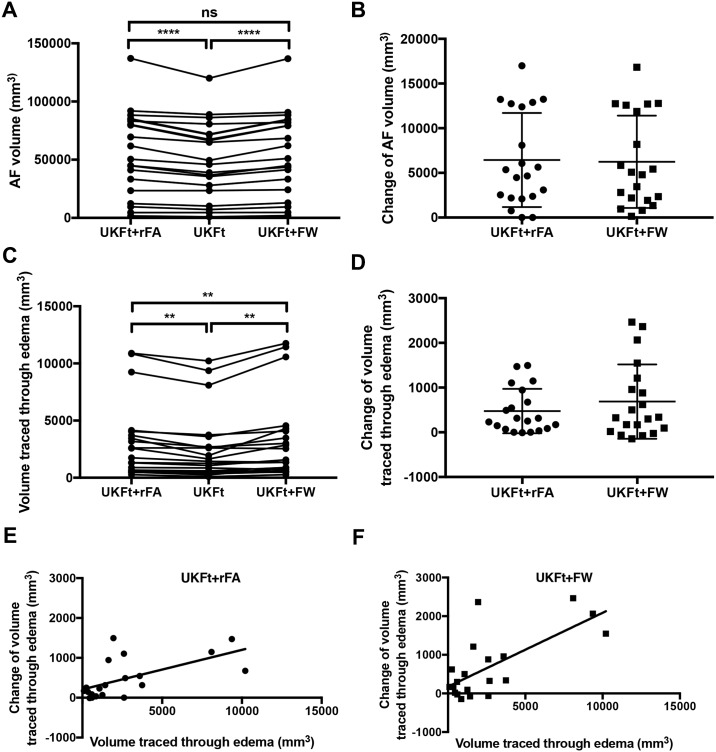
Graphs of the volumes of the AF in edematous hemispheres. (a) Significant differences between AF volumes (n = 20) using the UKFt and UKFt+rFA methods (p<0.0001) and using the UKFt and UKFt+FW methods (p<0.0001), two-tailed paired t-tests. No significant difference between the UKFt+FW and UKFt+rFA methods (p = 0.186), two-tailed paired t-test. (b) The volume changes of AF in edematous hemispheres with reduced FA threshold (UKFt+rFA) and with the addition of the FW model (UKFt+FW). (c) Significant differences between the volumes of the tract that intersected the edema using the UKFt and UKFt+rFA methods (p = 0.0004) and using the UKFt and UKFt+FW methods (p = 0.0015), two-tailed paired t-tests. A significant difference between the UKFt+FW and UKFt+rFA methods (p = 0.039), two-tailed paired t-test. (d) The volume changes of AF that intersected the edema with reduced FA threshold (UKFt+rFA) and with the addition of the FW model (UKFt+FW). (e) A significant correlation between the AF volume traced through edema using the UKFt+rFA method and the AF-edema intersection volume change using the UKFt vs UKFt+rFA methods, Pearson r = 0.695, p<0.0001. (f) A significant correlation between the AF volume traced through edema using the UKFt+FW method and the AF-edema intersection volume change using the UKFt vs UKFt+FW methods, Pearson r = 0.806, p<0.0001.

For the 20 edematous hemispheres of our patient dataset, the mean AF volume using the UKFt method was 49,942±32,488 mm^3^, while the mean AF volume using the UKFt+FW method was significantly higher at 56,183±35,527 mm^3^ (two-tailed paired t-test p<0.0001; Fig 6a and 6b). The mean AF volume using the UKFt+rFA method was similar to that using the UKFt+FW method (two-tailed paired t-test p = 0.186; Fig 6a and 6b), and was significantly higher than that using the UKFt method (two-tailed paired t-test p<0.0001; Fig 6a and 6b). The mean volume of the AF tract that intersected the edema using the UKFt method was 2,673±3,041 mm^3^, while the mean volume of the AF tract that intersected the edema using the UKFt+FW method was significantly higher at 3,360±3,670 mm^3^ (two-tailed paired t-test p = 0.0015; Fig 6c and 6d). The mean volume of the AF tract that intersected the edema using the UKFt+rFA method was also significantly higher than that using the UKFt method (two-tailed paired t-test p = 0.0004; Fig 6c and 6d); however, it was significantly lower than that using the UKFt+FW method (two-tailed paired t-test p = 0.039; Fig 6c and 6d). There was a significant correlation between AF volume affected by edema and the volume change with UKFt+FW (Pearson r = 0.806, p<0.0001) and the linear regression equation is "Y = 0.1824*X+73.89" (Fig 6e), as well as a significant correlation between AF volume affected by edema and the volume change with UKFt+rFA (Pearson r = 0.0.695, p<0.0001) and the linear regression equation is "Y = 0.1026*X+151.70" (Fig 6f). Comparing between the addition of the FW model (UKFt+FW) and the reduced FA threshold (UKFt+rFA), the UKFt+FW method had a higher correlation (Pearson r = 0.806) than the UKFt+FW method (Pearson r = 0.695).

**Fig 7:**
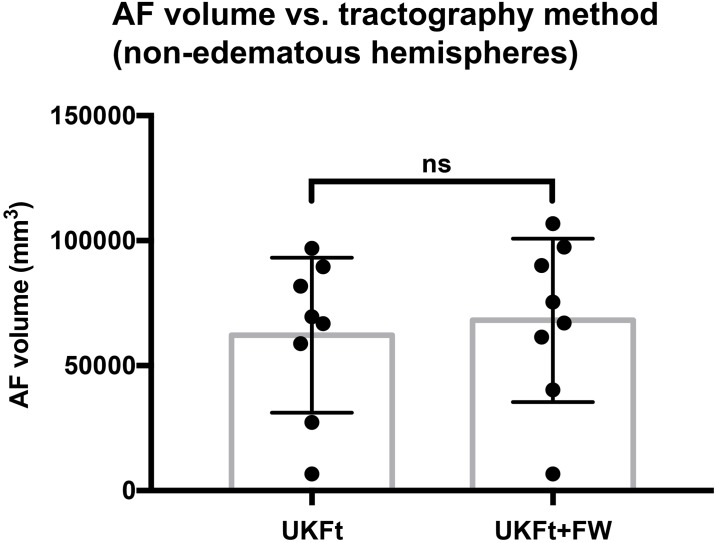
Graph of the volume of the AF in non-edematous tumor hemispheres. Bar graph shows no significant difference between AF volumes using the UKFt and UKFt+FW methods in nonedematous tumor hemispheres, n = 8, p = 0.0996, two-tailed paired t-test.

For the 8 tumor hemispheres of our patients without edema, there was no statistically significant difference between the UKFt and UKFt+FW volumes, where the mean AF volume using the UKFt method was 62,181±31,003 mm^3^ and the mean AF volume using the UKFt+FW method was 68,138±32,697 mm^3^, (two-tailed paired t-test p = 0.0996; Fig 7).

### 3.3. Effects of FW modeling on fiber number and length

**Fig 8:**
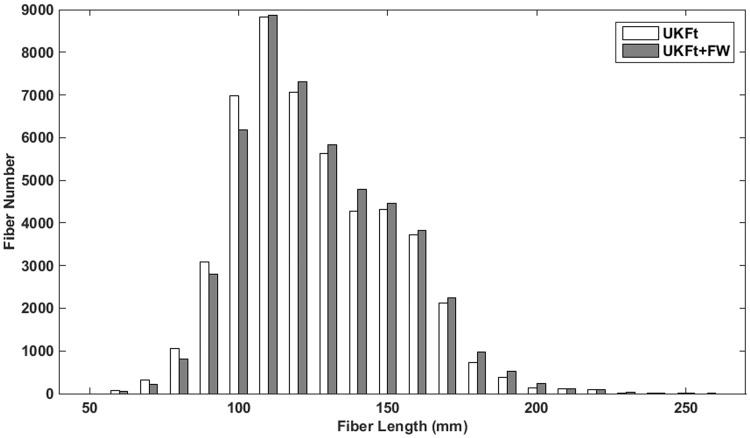
Histogram of fiber lengths in AF. Histogram shows the distribution of the number of fibers versus the fiber length of the AF tracts in all edematous hemispheres.

The number and the length of the fibers per AF tract in the edematous hemispheres were measured to assess differences in tractography performance between the UKFt and UKFt+FW methods. The mean numbers of fibers in AF were 2,540±2,907 and 2,571±2,899 using the UKFt and UKFt+FW methods, respectively. The mean lengths of fibers in AF were 127.8±9.9 mm and 130.4±9.9 mm using the UKFt and UKFt+FW methods, respectively. These increases in fiber number and length using the UKFt+FW method did not reach significance (p = 0.5883, p = 0.1630). In addition, we plotted the distribution of AF fiber length averaged over all edematous hemispheres in a histogram (Fig 8). The histogram shows that, compared to UKFt, the UKFt+FW method tended to track a larger number of long AF fibers (over 110 mm) but a smaller number of short fibers (under 110 mm).

### 3.4. Analysis of FW fraction for modeling edema

**Fig 9:**
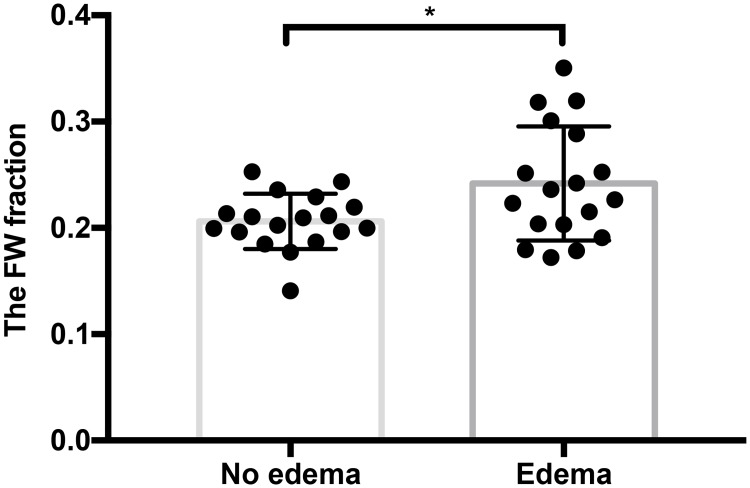
Mean FW fraction of AF fibers. Bar graph shows a significant difference between the mean FW fraction of fibers that traverse and did not traverse edema in the UKFt+FW method, n = 18 (data from 2 patients were excluded due to no fibers traversing no edema area), p = 0.0271, two-tailed paired t-test.

**Fig 10:**
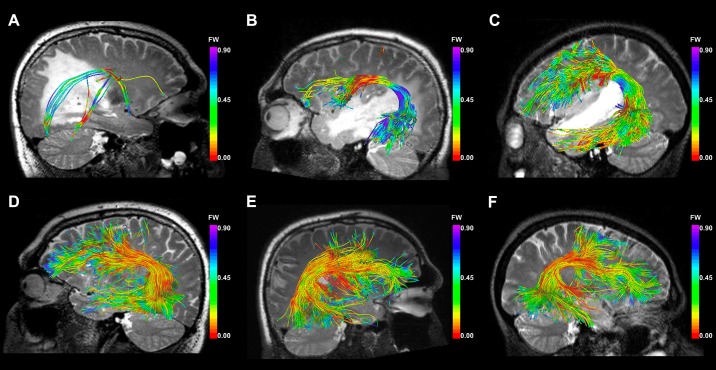
FW fraction of fibers near the tumor. Images show the fibers colored by the FW fraction near the tumor using the UKFt+FW method in edematous hemispheres of (a) P3, (b) P11, (c) P22 and non-edematous hemispheres of (d) P3, (e) P11, (f) P22. In these illustrative example cases, P3 has significant edema, P11 moderate edema, and P22 little edema affecting the AF. Along the fibers, the color scales are the same across the three patients: warm colors represent low FW fraction values, and cool colors represent high values (see color bar). A T2-weighted image is shown behind the fiber tracts.

The FW fraction is an estimate of the fractional volume of FW at each point along the tract, and thus it is expected to reflect the amount of edema encountered during fiber tracking. We measured the mean FW fraction of the fibers that traversed edema and those that did not traverse edema in the UKFt+FW method. The mean FW fraction of the fibers traversing edema was significantly larger (p = 0.0271) than that of the fibers that did not traverse edema (Fig 9). Fig 10 shows the FW fraction of fibers near the tumor in the three selected cases. It is visually apparent that the FW fraction in the part of the fibers intersecting the edema is higher than the FW fraction in the part of the fibers not intersecting the edema.

### 3.5. Expert judgment results

Expert judgment was performed on the AF tracts of the five patients (P3, P5, P6, P11, P19) that had the smallest AF volumes (972 mm3 to 23394 mm3) in the edematous hemisphere. In general, the three experts rated the three tracts based on whether they could identify the apparently true AF tracts near the edema while avoiding false positive/negative tracking. The averaged ranking scores of the UKFt, UKFt+rFA, and UKFt+FW methods were 1.67±0.62, 1.8±0.74 and 1.53±0.86, respectively, showing that the UKFt+FW method obtained the best expert evaluation performance in this experiment.

## Discussion

Our results demonstrated that the UKFt+FW method can trace a significantly larger volume of the AF than the UKFt method in the setting of peritumoral edema. The UKFt+FW method was assessed as having reconstructed a larger number of long fibers of AF. Edema was quantitatively modeled by an increased FW fraction in fibers traversing edema.

The results of the FW model were patient-specific: we found that in patients with a larger volume of AF tract affected by edema, the FW model produced a significantly greater increase in the volume of the tractography. This was also seen in the illustrated cases (P3 with significant edema, P11 with moderate edema, and P22 with little edema affecting the AF). In P3, tracts were visually larger using the UKFt+FW method, while in P11 tracts were subtly larger and in P22 tracts were visually almost the same as the UKFt method. In addition, in patient P11, the trajectory of the fibers subtly changed when using the UKFt+FW method. Expert evaluation of the AF tracts obtained by different methods showed that experts generally preferred the AF tract obtained by using the FW model, according to their anatomical knowledge and considering the potential influence of the final results on the surgical route. This experiment focused on a patient subset with the lowest AF volumes, i.e. those relatively highly affected by edema. While these initial results showed that UKFt+FW received the best (lowest) mean expert rating, a larger patient dataset would be required to encounter a sufficient sample of low-volume AFs to assess statistical significance of expert preferences.

More research is still needed to validate the performance of the UKFt+FW method. To better understand the clinical implications of using the FW model, future investigations could include other fiber tracts and multiple patients with different levels of edema, locations of tumor, relationships between tract and tumor, and surgical outcome correlation. We note that there are other mathematical methods that can model free-water diffusion in dMRI, for example the isotropic volume fraction in the neurite orientation dispersion and density imaging (NODDI) framework [51–53]. Fig 10 demonstrates that the AF FW fractions measured in the healthy contralateral hemispheres are in good numerical correspondence with the isotropic fraction from the NODDI model, which we previously measured in the AF using UKFt from high-quality Human Connectome Project healthy subject data [54]. Both methods produce values of 0.1 to 0.2 in the body of healthy AF with locally higher values nearing the cortex. Future work could compare such models with the results of the FW model in the context of peritumoral edema. We believe that such empirical experiments are relevant clinically, both to potentially reduce user interaction for selecting tracking thresholds and to raise awareness of potential modeling strategies for edema. However, it is important to state that we currently lack ground truth regarding the existence of a free water compartment and how it may change in edema. While we have performed initial experiments demonstrating that UKFt+FW can recover fiber tracts in a synthetic edema phantom [22], modeling of edema is a challenge. It is clear that edema results in an increase in mean diffusivity (isotropic diffusion) and in free water [12], but it is well-known that due to the unique nature of each brain tumor, the specific diffusion properties of peritumoral edema vary across patients and tumor types [55,56], with additional patient-specific local variability depending on the distance from the tumor [57]. For these reasons it is likely impossible to come up with optimal tractography parameters for the whole brain because every patient and tumor is different, and edema also varies spatially within a single patient. Thus our experiments in the current work aim to assess if a locally adaptive model of edema can somewhat ameliorate the need for varying multiple tractography threshold parameters.

The performance of the FW model in our study is partly due to its ability to increase the length of fibers traversing edema. This can be explained by the fact that using a FW model is similar to employing a locally adaptive stopping threshold for fiber tracking. As the FW tensor model is spherical, it can improve the fiber model fits by decreasing the influence of edema without impacting the estimation of fiber orientations [12]. By separately modeling the isotropic FW, the UKFt+FW method may model higher anisotropy of the fiber being tracked. A longer fiber length may therefore be achieved before reaching the threshold that stops fiber tracking. The most clinically used threshold is FA, which corresponds to the DTI model. In our experiments, we evaluated the tracts computed using a reduced FA threshold (UKFt+rFA) in both quantitative and qualitative comparisons. As expected, using a lower FA threshold could increase the volume of the tracked AF tract, as well as the volume traced through edema. However, in comparison with the UKFT+FW model, the UKFT+rFA method was less effective in tracking through the edema, as suggested by the fact that the UKFT+rFA method had a significantly lower volume of AF traced through edema than the UKFt+FW method (Fig 6c and 6d).

Currently, manual patient-specific interactive adjustment of the FA threshold is the standard clinical approach to increase sensitivity for tracking through edema [18]. However, more advanced diffusion models than the diffusion tensor have many different model-specific threshold parameters, and it is possible for a single model to have multiple complementary thresholds used for stopping. Expert setting of parameters is currently the gold standard for neurosurgical planning brain mapping tasks such as tractography seeding [18] and fMRI thresholding [58,59]. While it is possible that expert interaction provides the best parameter settings on a patient-specific basis, it is known that there is variability across experts, who may not agree on the best parameter settings [23,24]. In the interest of automating tractography for surgical planning [16,60] to avoid time-consuming and operator-dependent expert tract selection [23,61], it is important to develop locally adaptive and automated criteria to enable fiber tracking through edema without user interaction. The addition of a FW model is one possible way to address this need. Recent research has proposed sophisticated rules for tracing fibers according to anatomical constraints (such as a tract should end in gray matter) to reduce bias in tractography [62,63]. This type of automated tractography filtering has promise for the future in neurosurgical planning. However, these rules rely on automated brain segmentation, which is not yet robust in the presence of brain tumors.

To our knowledge, the present study is the first one to apply a FW model to a retrospective cohort of patients with brain tumors and to quantitatively compare to tractography without FW. So far, to our knowledge one other group has investigated a FW model in tractography of neurosurgical patients. Their results, which indicated good performance in a test of a FW model in five patients but did not include a comparison to any other tractography methods [32,33], were presented as part of the international DTI Challenge [64].

In related work, many multi-fiber methods have been shown to improve tractography in patients with brain tumors, including spherical deconvolution [7], diffusion tensor with probabilistic tracking [65], generalized q-sampling imaging (GQI) [9], high-definition fiber tractography (HDFT) [66], q-ball fiber tractography [67], and multi-tensor UKFt [14,15]. Similar to our current study, these related tractography comparison studies have considered that the goal of increased sensitivity, in the sense of detecting more or larger neuroanatomically plausible fiber tract structures in the clinically critical peritumoral area, is beneficial for neurosurgical planning [7,9,15,65,68]. This is motivated by the clinical observation that patients with some intact language and/or motor function must have preserved functional fiber tracts, despite the changes in the diffusion MR signal due to infiltration and/or edema that could prevent complete tract tracing. For instance, Kuhnt et al. applied high angular resolution diffusion imaging (HARDI) with compressed sensing-based tractography and found it produced visually improved tractography in 6 brain tumor patients in the AF compared with DTI-based results [68]. Zhang et al. showed that using GQI they could visualize pyramidal tracts (4 patients) and ventral language-related tracts (1 patient) in the setting of edema better than DTI [9]. A recent study by Caverzasi et al. applied HARDI q-ball tracking in presurgical planning for language pathways, with a focus on prediction of long-term language dysfunction [67]. While these studies showed the benefits of multi-fiber tractography, they did not incorporate a mathematical model of edema.

There are challenges in the interpretation of tractography in or near peritumoral edema. As in our previous study comparing UKFt and DTI tractography in AF [15], here we found a relatively large variability in the size of AF across neurosurgical patients. Even in healthy individuals, this structure has significant variability in size and asymmetry [50,69]. In patients, fibers that are apparently “absent” on tractography may not be destroyed by tumors, particularly for those patients without apparent functional impairment [15,70]. Therefore, increasing the sensitivity for tracking critical functional fiber tracts can be important for neurosurgical planning. We previously showed that our atlas-based automated method was more robust than expert tract selection in AF, in the sense that the automated method detected larger structures that better intersected patient-specific language fMRI as anatomically expected [16]. However, recent studies indicate that advanced multi-fiber tractography methods may reduce false negatives at the expense of increased false positives [71,72]. Though length and volume measures are widely used to quantitatively assess tractography [14,15,72,73], false positives are an important issue in tractography [71,72] and could confound these quantitative measures. To ameliorate this issue to some extent, we have included visualizations for comparison across methods and judgments from expert raters. Our results indicate that the UKFt+FW method provides the ability to track a larger volume AF (Fig 6) with more plausible fibers according to expert assessment in the setting of peritumoral edema. Although this result could lead the neurosurgeon to resect less tissue and perform a more conservative surgery, it is considered best for neurosurgeons to have the most information possible and then determine the value of this information to make a clinical judgment [15,73,74].

Although this study shows potential advantages regarding the UKFt+FW method, certain limitations of the study should be mentioned. First, echo-planar imaging (EPI) distortion is a clinical challenge in dMRI [75]. Second, tractography methods are under active development and evaluation, with many competing algorithms to choose from, and there remains significant anatomical controversy about the true extent and termination of many fiber tracts in the human brain [16,76]. Technical challenges for tractography include resolving crossing versus kissing fibers [77] and increased false positives with multi-fiber models [71]. Thus, there is unavoidable uncertainty in these and any other tractography results. Third, there are many threshold parameters that can be used to start and stop tractography, and it is possible to increase the size of a fiber tract by reducing these thresholds. In a recent study, we exhaustively varied multiple complementary and interacting threshold parameters in a limited cohort of two tumor patients, finding that the generalized anisotropy threshold had a larger effect on UKFt than the addition of a FW model [22]. The results of the current study, in a much larger cohort, indicate that the addition of a FW model has the largest effect in those patients where the fiber tract is most affected by edema. Fourth, we have chosen to report results in terms of the tract volume as in [14,15] since it has a clearer biological interpretation than the “fiber count”, which is a count of streamlines produced by tractography and not directly related to the number of axons [78]. Our implementation of volume measurement carefully upsamples points along each fiber to ensure an accurate measure of all voxels through which the fiber passes; however, it is true that the tract volume measure can be sensitive to relatively small increases in the numbers of fibers or their length. Finally, the relatively high b (2000) single-shell protocol was not optimized for the estimation of FW. Future studies could use multi-shell protocols combining both higher b-values for optimized tractography with lower b-values for improved free water estimation.

Overall, our results indicate that biophysical models of edema can increase the sensitivity of tractography (in the sense of tracking a larger volume of fibers) in regions of peritumoral edema in patients with brain tumors. This result has importance because most intra-axial lesions are metastases and high grade gliomas, which are associated with peritumoral edema. The assessment of which combination of acquisition strategy, fiber model, and tractography method performs the best for neurosurgical planning remains an open question for future research.

## Supporting information

S1 Dataset Supporting information: Measured data. This file includes the data presented in the paper.
(XLSX)
